# A critical epitope in CD147 facilitates memory CD4^+^ T-cell hyper-activation in rheumatoid arthritis

**DOI:** 10.1038/s41423-018-0012-4

**Published:** 2018-03-21

**Authors:** Na Guo, Sheng Ye, Kui Zhang, Xiaoling Yu, Hongyong Cui, Xiangmin Yang, Peng lin, Minghua Lv, Jinlin Miao, Yang Zhang, Qing Han, Rongguang Zhang, Zhinan Chen, Ping Zhu

**Affiliations:** 1 Department of Clinical Immunology, Xi Jing Hospital, Xi’an, ShaanXi 710032 P.R. China; 2 Department of Cell Biology, National Translational Science Center for Molecular Medicine, The Fourth Military Medical University, Xi’an, ShaanXi 710032 P.R. China; 3 National Laboratory of Biomacromolecules, Institute of Biophysics, Chinese Academy of Sciences, Beijing, 100101 P.R. China; 4 National Center for Protein Science Shanghai, Institute of Biochemistry and Cell Biology, Shanghai Institutes for Biological Sciences, Chinese Academy of Sciences, Shanghai, 201203 P.R. China

**Keywords:** CD4^+^ Memory T cell, CD147, Monoclonal Antibody, Rheumatoid arthritis, Immunotherapy

## Abstract

The abnormal activation of CD4^+^CD45RO^+^ memory T (Tm) cells plays an important role in the pathogenesis of rheumatoid arthritis (RA). Previous studies have shown that CD147 participates in T-cell activation. However, it remains unclear whether CD147 is involved in abnormal Tm-cell activation in RA patients. In this study, we demonstrated that CD147 was predominantly upregulated in Tm cells derived from RA patients. The anti-CD147 mAb 5A12 specifically inhibited Tm-cell activation and proliferation and further restrained osteoclastogenesis. Using a structural–functional approach, we depicted the interface between 5A12 and CD147. This allowed us to identify two critical residues, Lys63 and Asp65, as potential targets for RA treatment, as the double mutation K63A/D65A inhibited Tm-cell activation, mimicking the neutralization by 5A12. This study provides not only a theoretical basis for a “CD147-Tm/Osteoclast-RA chain” for the potential prevention and treatment of RA or other T-cell-mediated autoimmune diseases but also a new target for related drug design and development.

## Introduction

Rheumatoid arthritis (RA) is one of the most common inflammatory rheumatic diseases and is heterogeneous with a complex and yet not fully understood mechanism. It is characterized by joint inflammation, progressive joint destruction, and increasing disability.^1^ In past years, considerable therapeutic treatments have been used and functioned either by blocking pro-inflammatory cytokines or targeting cells that are closely involved in the pathophysiology of RA.^2^ T cells, especially CD4^+^ T cells, are supposed to play a central role in the development and progression of RA. Activated T cells can secrete various cytokines and subsequently activate innate immune cells, support B-cell activation, and induce destructive chondrocyte and osteoclast activation.^3^ Thus, strategies targeting T cells were applied to limit and down-modulate T-cell-mediated autoimmune diseases. The anti-CD3 antibody OKT3 has been used successfully to treat acute rejection after allogeneic organ transplantation. However, this antibody can induce severe cytokine release syndrome.^4^ Another agent known as abatacept, a human CTLA4–IgFc fusion protein, prevents the delivery of the second co-stimulatory signal required for the optimal activation of T cells.^5^ Nonetheless, due to the broad inhibition of all T cells to prevent autoimmune attacks, the chances of infection also increase. Therefore, the development of immunomodulators, preferably specific cell-targeting approaches, might lead to treatments with an improved pharmacological safety profile and a lower incidence of adverse effects.

Previous studies have demonstrated increased numbers of activated CD69^+^CD4^+^ T cells in the peripheral blood and augmented infiltration in the synovial tissue of RA patients.^6, 7^ Interestingly, the majority of these accumulated activated CD69^+^CD4^+^ T cells in the synovial fluid (SF) were memory T (Tm) cells,^8^ indicating the continuous hyper-activation of Tm cells in RA patients, although the different factors responsible for this elevation are not satisfactorily understood. Tm cells, due to their rapid and robust responses upon antigen recognition, were thought to be much more pivotal in mediating the persistence of autoimmune diseases than naive T (Tn) cells.^9^ It has also been shown that inflammatory cytokines, such as interleukin-1α (IL-1α), IL-2, IL-6 and tumor necrosis factor-α (TNF-α), are generally abundant in synovial tissue and fluid from patients with RA, which were produced during RA progression by activated Tm cells rather than CD4^+^ Tn cells.^10^ The continuous activation of Tm cells could be the etiology of RA development, which could result in the unwanted activation of other joint infiltrating cells, including macrophages, fibroblasts, B cells, and dendritic cells, and a further increase in the secretion of inflammatory cytokines and chemokines, leading to joint synovitis and cartilage and bone erosion.^11^ All these facts suggest that the abnormal activation of Tm cells plays a critical role in the pathogenesis of RA, and a strategy specifically designed to target Tm cells might be a promising therapy for RA treatment. However, the mechanisms of the regulation of Tm-cell activation are still not fully understood.

CD147 is a highly glycosylated transmembrane protein that belongs to the immunoglobulin superfamily and has been found to have multiple roles in physiological and pathological functions, such as cell migration, invasion, adhesion, and energy metabolism.^12–14^ Early studies have demonstrated a close association between CD147 and T-cell activation and proliferation. In 1992, CD147 was originally identified as a T-cell activation-associated antigen, named M6 based on phytohemagglutinin-activated T lymphocyte experiments.^15^ Later studies further confirmed that CD147 is expressed weakly in resting T lymphocytes and that its expression rapidly increases upon activation.^16^ In recent years, increasingly more studies have focused on determining how CD147 contributes to the pathogenesis of autoimmune diseases, given the potential correlation between CD147 and immune-based inflammatory diseases mediated by the abnormal activation of various T-cell subsets.^17, 18^ Interestingly, some prototypes of autoimmune diseases characterized by T lymphocyte dysfunctions, such as systemic lupus erythematosus (SLE) and RA, were reported to exhibit high expression of CD147 on T cells in both the peripheral blood and local inflammatory lesions,^19, 20^ suggesting a potential role for CD147 in autoreactive T-cell activation. Our previous studies investigated the expression of CD147 on different immune cells, including neutrophils, monocytes, macrophages, and synoviocytes from RA patients, and demonstrated that all of these cells expressed high levels of CD147.^21–23^ Furthermore, blocking CD147 with a specific antibody inhibited the induction of matrix metalloproteinases and angiogenesis in RA. However, the exact roles and mechanisms of CD147 in the T-cell-mediated pathogenesis of RA are still not fully understood. Exploring the full impact of CD147 on the Tm response and Tm-mediated effects is vital to a better understanding of the mechanisms of autoimmunity and will provide a new potential target for regulating immune responses.

In the present study, we demonstrated that the expression of CD147 was significantly increased on the Tm-cell surface in RA patients. Silencing of CD147 expression on Tm cells resulted in significant inhibition of Tm-cell activation. The anti-CD147 mAb 5A12, which was developed by our group, was found to selectively inhibit Tm-cell activation and proliferation and Tm-mediated osteoclastogenesis by the suppression of ZAP70–LAT–ERK signaling. In addition, we resolved the crystal structure of the CD147-5A12 Fab complex, discovered the binding epitope and determined which residues are critical for the co-stimulatory function of CD147.

## Results

### Activated CD4^+^ Tm cells accumulated in both SF and PBMCs derived from RA patients

**Fig. 1:**
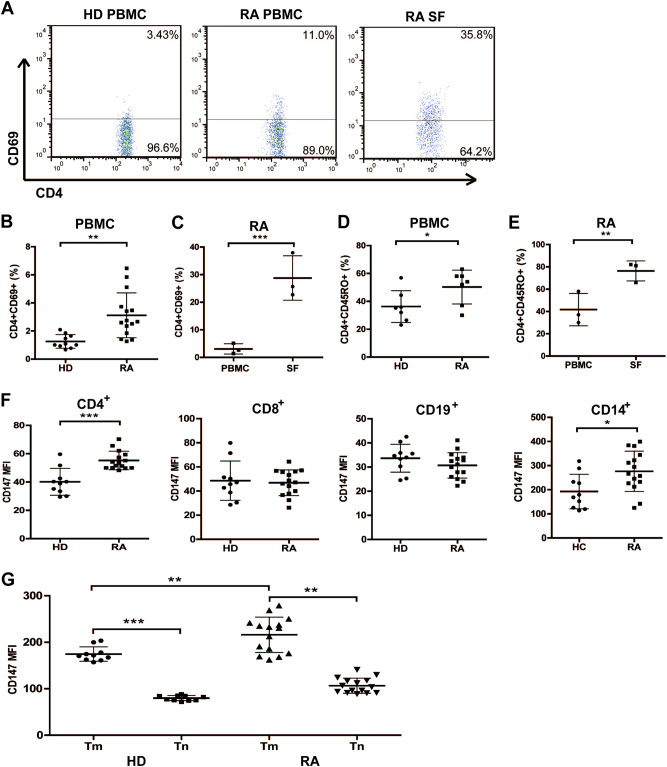
RA patients exhibit a higher number of Tm cells with high expression of CD147. **a** Representative FACS data show the expression of CD69 on CD4^+^ T cells in PBMCs from healthy donors (HD) and RA patients, and three samples of SF from the latter were detected. **b** Summary data of CD69 expression on CD4^+^ T cells in PBMCs from HD (*n* = 10) and RA patients (*n* = 15). Each symbol represents a value from a single donor. The horizontal lines denote the mean ± SD expression. **c** Summary data of CD69 expression on CD4^+^ T cells in PBMCs and SF from RA patients from three independent experiments are shown. **d** Detection of CD4^+^CD45RO^+^ T cells in PBMCs from HD (*n* = 7) and RA patients (*n* = 7). **e** Detection of CD4^+^CD45RO^+^ T cells in PBMCs and SF from RA patients (*n* = 3). **f** CD147 expression on different peripheral blood cells from HD and RA patients was determined by FACS. **g** Expression of CD147 on Tm and Tn cells in both RA patients and HD. *P < 0.05; **P < 0.01; ***P < 0.001

Since there is growing evidence for the participation of activated T cells in RA, we first assessed the surface expression of CD69 on CD4^+^ T cells from both RA patients and healthy donors (HD). The percentage of CD69^+^ activated CD4^+^ T cells in the peripheral blood mononuclear cells (PBMCs) in RA patients was significantly higher than that in HD group (Figs. 1a, b). Moreover, higher levels of CD69 expression on CD4^+^ T cells from SF than from PBMCs were also observed in RA (Fig. 1c). The number of Tm cells was increased in the CD4^+^T-cell population of the peripheral blood (Fig. 1d). Moreover, the great majority of Tm cells was present in SF (Fig. 1e), indicating that the accumulation of Tm cells is a common phenomenon during local inflammatory responses in RA. Therefore, more activated Tm cells exist in RA patients than in HD, which provides further support for the hypothesis that Tm cells play an important role in RA pathogenesis.

### CD147 is highly upregulated on Tm cells from RA patients

Although CD147 was broadly expressed on human peripheral blood cells, its detailed roles and the contribution of different cell subsets to RA pathogenesis have not fully been addressed. We verified and further compared the CD147 expression profiles in different cells from RA patients and HD. Fluorescence-activated cell sorting (FACS) analyses showed that CD147 expression is different on CD14^+^ monocytes, CD19^+^ B cells and CD4^+^ and CD8^+^ T cells. Interestingly, the expression of CD147 on circulating CD4^+^ T cells was much higher in RA patients than in HD (Fig. 1f), and a significantly higher expression of CD147 was observed on Tm cells than on Tn cells in both RA patients and HD. It is also interesting that CD147 expression on Tm cells is much higher in RA patients than in HD (Fig. 1g).

Tm cells in humans have classically been subdivided into two groups: central memory T (Tcm) cells and effector memory T (Tem) cells. We also tested CD147 expression in both the Tcm and Tem cell subsets. We demonstrated that CD147 expression showed no significant difference between Tcm and Tem cells in either HD or RA patient PBMCs. Additionally, there was no significant difference between Tcm and Tem cells in RA SF. Furthermore, the proportions of Tcm and Tem cells showed no significant differences in either HD or RA patient PBMCs and RA SF (Figure S1).

### mAb 5A12 specifically inhibits Tm-cell activation and proliferation but has no effect on Tn cells derived from RA patients

**Fig. 2:**
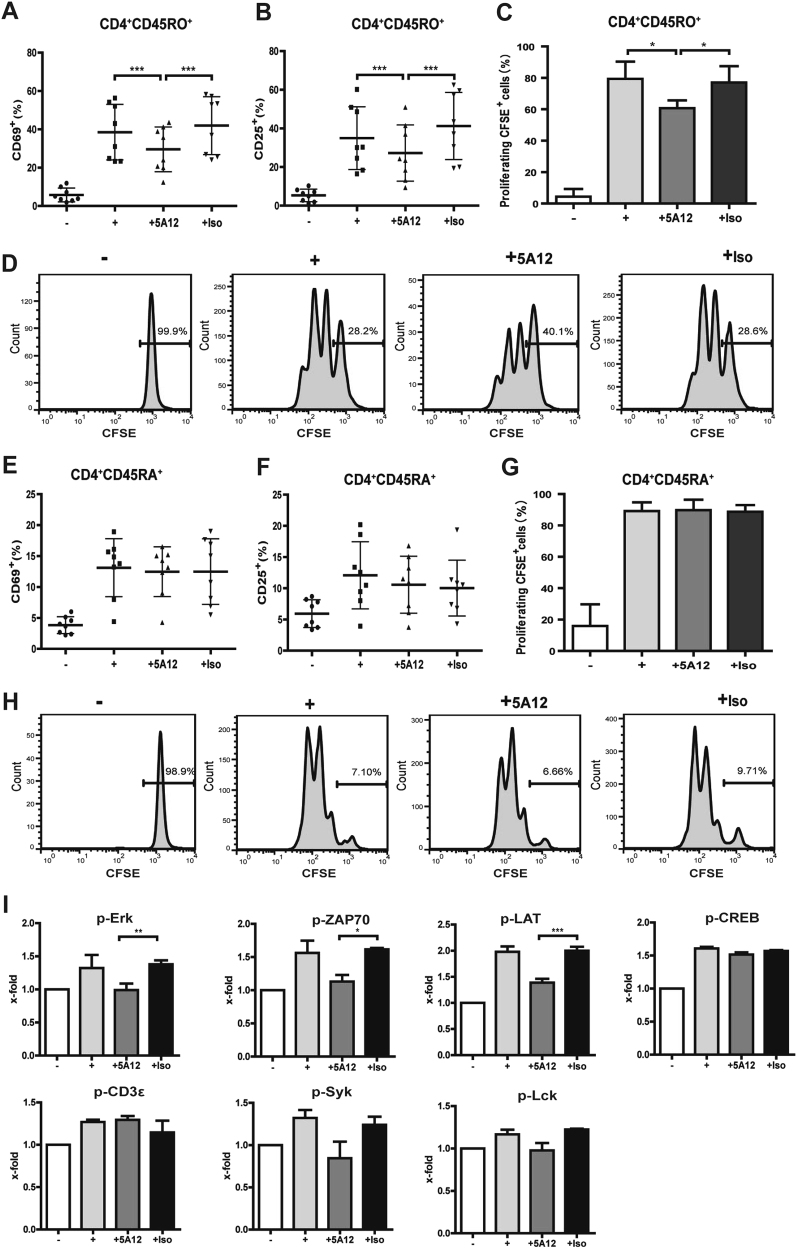
Tm-cell activation and proliferation is significantly inhibited by CD147 mAb 5A12. **a**, **b** CD147 engagement by 5A12 resulted in the downregulation of TCR-induced CD69 and CD25 expression on Tm cells from RA patients (*n* = 8). The expression of CD69 at 6 h and CD25 at 24 h following activation of purified CD4^+^CD45RO^+^ T cells by anti-CD3/CD28 mAbs with or without 5A12 treatments was determined. **c** In vitro, 5A12 inhibited Tm-cell proliferation induced via TCR engagement by anti-CD3/CD28 mAbs for 72 h, as determined by CFSE staining and FACS. **d** Representative data from one of three experiments are shown. Histograms were gated on the Tm population. **e**-**h** Similar experiments were performed to observe the effects of 5A12 on Tn cells. **i** Effects of 5A12 on phosphorylation of T-cell activation by Luminex analyses. *P < 0.05; **P < 0.01; ***P < 0.001

We previously reported a panel of newly established anti-CD147 mAbs and found that mAb 5A12 could inhibit CD3–T-cell antigen receptor (TCR)-stimulated CD4^+^ T-cell activation and proliferation. In this article, we examined whether the suppressing effect of 5A12 is confined to different T-cell subsets. As shown in Figs. 2a, b, expression of both activation markers (CD69 and CD25) in resting Tm cells was low. Upon activation, Tm cells exhibited markedly upregulated expression of the cell surface molecules CD69 and CD25. When treated with 5A12, upregulation of both CD69 and CD25 was significantly inhibited. We also demonstrated that 5A12 inhibits Tm-cell activation in a time-dependent manner (Figure S2). We further investigated whether 5A12 influenced TCR-dependent T-cell proliferation. Figs. 2c, d show that Tm-cell proliferation was significantly inhibited by 5A12 compared with an isotype control. As shown in Figs. 2e-h, 5A12 exhibited no inhibitory effects on the activation markers CD69 and CD25, as well as proliferation of Tn cells.

Mounting evidence shows that RA patients produce abundant pro-inflammatory cytokines, which play an indispensable role in RA pathogenesis. Therefore, we harvested human serum from both RA patients and HD to determine the production of various cytokines by Luminex. Higher levels of IL-6 and interferon (IFN)-γ were detected in serum from RA patients, and treatment with 5A12 resulted in a significant decrease of IL-6, TNF-α and IL-17A production (Figure S3).

### mAb 5A12 inhibits TCR-induced protein tyrosine phosphorylation in Tm cells

The mechanisms underlying the suppression of Tm-cell activation by 5A12 were further elucidated by a Luminex flow fluorescence technique (Liquid chip). As shown in Fig. 2i, the levels of p-ZAP70, p-Eek, p-CREB, and p-LAT were significantly increased upon stimulation by anti-CD3/CD28 mAbs. However, pretreatment with 5A12 significantly lowered the levels of p-ZAP70, p-LAT and p-Erk compared with that in the isotype controls.

### mAb 5A12 specifically inhibits activated Tm-mediated osteoclastogenesis in vitro

**Fig. 3:**
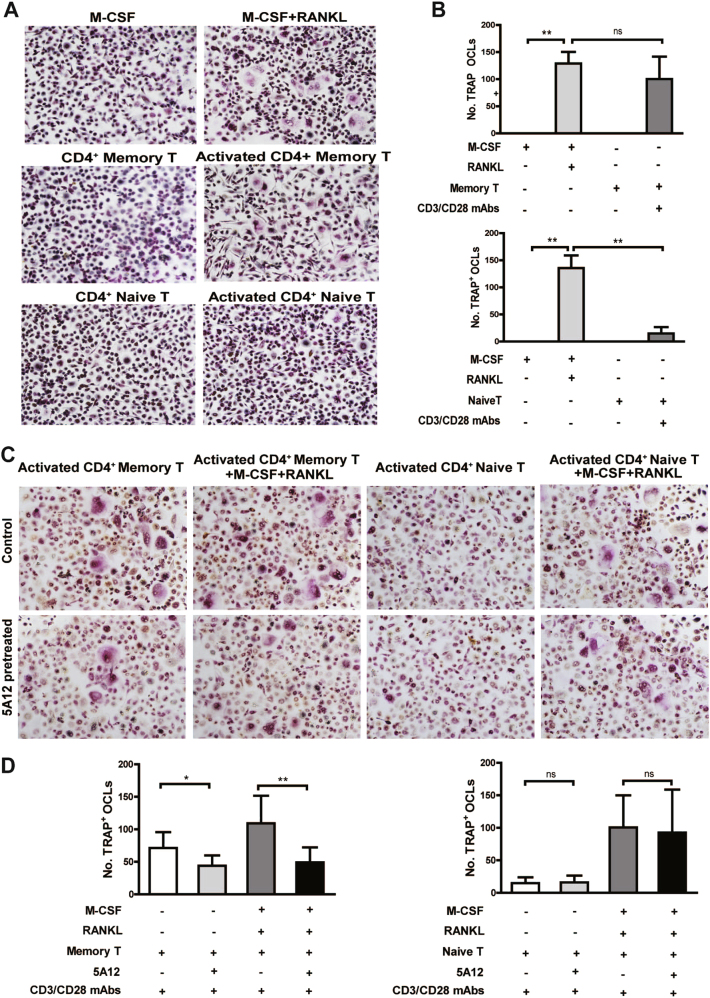
Effect of mAb 5A12 on activated Tm-mediated osteoclastogenesis. **a**, **b** Enhanced osteoclastogenesis by activated Tm cells in a co-culture system but not by activated Tn cells. The corresponding statistical results for TRAP^+^ cells are shown. **c**, **d** Effects of 5A12 on Tm-mediated osteoclastogenesis. 5A12 could significantly inhibit Tm-mediated osteoclastogenesis in the presence or absence of rhM-CSF and RANKL. The corresponding statistical results of TRAP^+^ cells are shown. *P < 0.05; **P < 0.01; ns, not significant

In RA, bone erosion is a critical pathology characterized by increased osteoclastogenesis. Activation of CD4^+^ T cells has been reported to be linked to this pathological progress; however, whether Tm induces osteoclast formation has not been determined. Interestingly, treatment with activated Tm cells under anti-CD3/CD28 mAbs stimulation increased the number of differentiated osteoclasts as much as macrophage colony-stimulating factor (M-CSF) and receptor activator of nuclear factor κB ligand (RANKL) did, whereas resting Tm cells alone failed to induce osteoclastogenesis, as M-CSF did (Figs. 3a, b). However, for Tn cells, neither an activated nor a resting state induced monocytes to differentiate into mature osteoclasts. Because CD147 participates in Tm-cell activation, we further investigated whether CD147 is involved in Tm-mediated osteoclastogenesis. Both Tm and Tn cells were pretreated with 5A12 before co-culturing with CD14^+^ monocytes. As shown in Figs. 3c, d, 5A12 significantly inhibited in vitro osteoclast differentiation in a co-culture of activated Tm cells and monocytes supplemented with either blank or RANKL and M-CSF; however, 5A12 exhibited no inhibitory effect on Tn-mediated osteoclastogenesis.

### Silencing CD147 suppresses Tm-cell activation

**Fig. 4:**
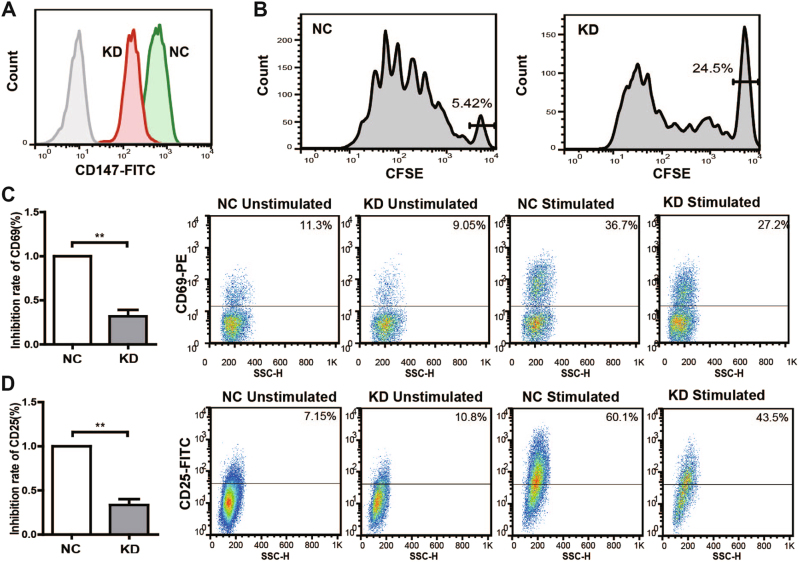
Downregulation of CD147 inhibits Tm-cell activation and proliferation. **a** FACS analyses showed the suppression of CD147 expression on Tm cells via lentiviral interference. The black lines represent the negative control memory T cells (NC), and the red lines represent the CD147 knockdown of the T cells (KD). **b** Knockdown of CD147 significantly inhibited Tm-cell proliferation in vitro induced via TCR engagement by anti-CD3/CD28 mAbs for 120 h, as determined by CFSE staining and FACS. **c**, **d** Knockdown of CD147 resulted in a prominent decrease of CD69 in vitro induced via TCR engagement by anti-CD3 mAb for 6 h and of CD25 via TCR engagement by anti-CD3/CD28 mAbs for 24 h on Tm cells. The corresponding statistical results for the inhibition rate and representative data are shown. **P < 0.01

Because the functional outcome of quantitative CD147 expression on Tm cells was not clearly assessed, we examined the effect of CD147 on Tm-cell activation using a lentiviral vector. As shown in Fig. 4a, FACS analyses revealed decreased protein expression of CD147 on lentivirus-transfected Tm knockdown (KD) cells relative to the control (NC). Interestingly, the knockdown of CD147 inhibited the activation of Tm cells by downregulating CD69 and CD25 expression (Figs. 4c, d). Moreover, the downregulation of CD147 also suppressed cell proliferation in vitro (Fig. 4b).

### Crystal structure of the CD147_ecto_-5A12_Fab_ complex reveals the 5A12 epitope

**Fig. 5:**
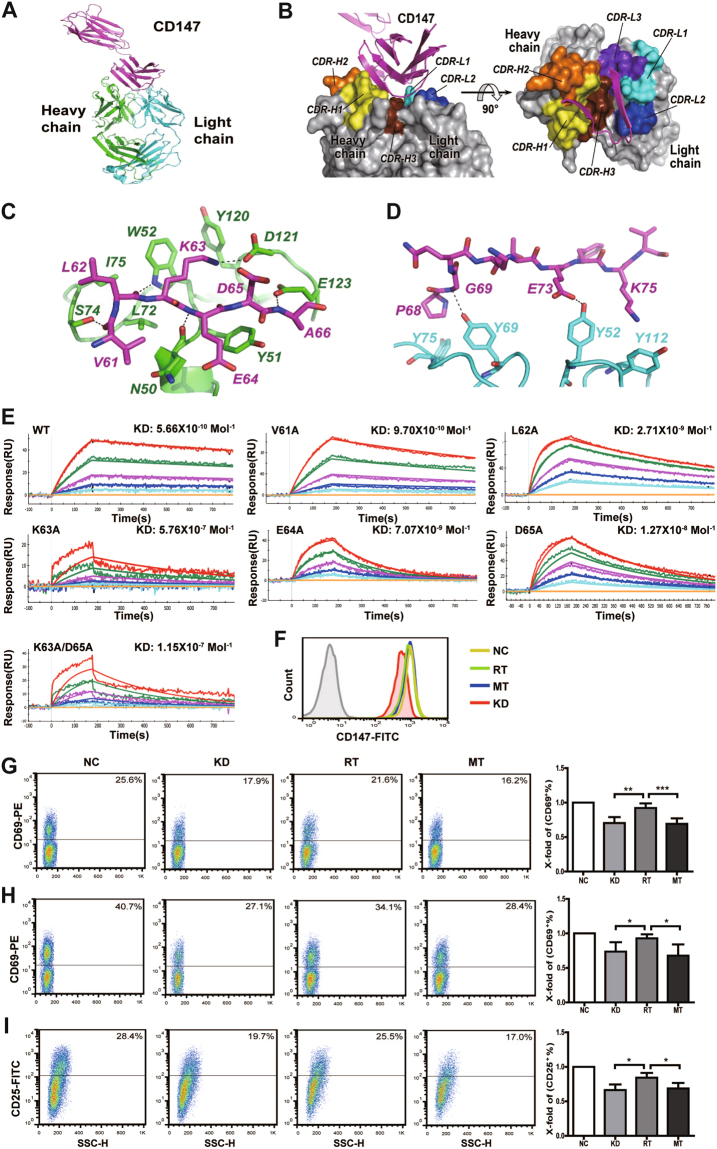
Crystal structure of the CD147_ecto_-5A12_Fab_ complex and function test of CD147-K63A/D65A mutation type. **a** Overall structure of the CD147-5A12 complex. The ribbon represents the CD147 ectodomain (magenta) and 5A12 Fab (heavy chain, green; light chain, cyan). **b** The CD147 footprint on 5A12. The 5A12 Fab structure (gray) is rendered to show the surface features that contribute to its idiotope by its CDRs. Both the heavy and light chains are involved in the interactions with CD147. In a head-on view with respect to the idiotope rotated 90°, a groove is formed among CDR-H1, -H2 and -H3. **c** The interactions between CD147 and the 5A12 heavy chain. Stick representations of the βC’ of CD147-D1 (carbon, magenta; nitrogen, blue; oxygen, red) and the heavy chain residues of 5A12 (carbon, green; nitrogen, blue; oxygen, red) interacting with it. Hydrogen bonds are indicated as dashed black lines. **d** The interactions between CD147 and the 5A12 light chain. Stick representations of the βE of CD147-D1 (carbon, magenta; nitrogen, blue; oxygen, red) and the light chain residues of 5A12 (carbon, cyan; nitrogen, blue; oxygen, red) interacting with it. **e** Representative sensorgram for 5A12 and CD147 interaction. **f** Identification of CD147 expression in transfected cells. CD147 expression was significantly downregulated in the KD group compared with the negative control (NC); rescue of CD147 mutant (MT) and revertant type (RT) was performed by RNAi-resistant gene re-expression. **g** CD147-K63A/D65A mutation resulted in a prominent decrease of CD69 expression on memory T cells upon single anti-CD3 stimulation for 6 h. **h**, **i** CD147-K63A/D65A mutation resulted in a prominent decrease of both CD69 and CD25 expression on memory T cells upon double anti-CD3/CD28 stimulation for 24 h. The corresponding statistical results of the inhibition rate and representative data are shown. *P < 0.05; **P < 0.01; ***P < 0.001

To identify the epitope of mAb 5A12 on CD147, as well as the key residues at the interface, we co-crystallized the ectodomain of CD147 (CD147_ecto_) and the Fab fragment of 5A12 (5A12_Fab_) and then determined the crystal structure of the CD147_ecto_-5A12_Fab_ complex at a resolution of 2.6 Å, which is the first structure of CD147 reported in complex with an anti-CD147 mAb. In the final model, each 5A12_Fab_ binds to one CD147_ecto_ at the flank of its N-terminal domain (D1), which is an IgC2-type immunoglobulin-like domain, revealing the epitope for 5A12, which mainly comprises two adjacent β-strands of CD147-D1, βC’ and βE, and the loop connecting them (Fig. 5a). The CD147 footprint on 5A12 involves all the three complementarity-determining regions (CDRs) on the heavy chain (CDR-H1, -H2 and -H3) and two CDRs on the light chain (CDR-L1 and -L2) (Fig. 5b), burying a total of 749 Å2 at the CD147_ecto_-5A12_Fab_ interface (455 and 294 Å2 for the heavy and light chains, respectively), which is approximately 8% of the entire surface area of CD147_ecto_.

The CD147 binding interface of the heavy chain of 5A12 is located at a groove formed by CDR-H1, -H2 and -H3, and D1-βC’ (61VLKEDA66) is half buried in the groove. To form the groove, CDR-H2 is extended longer than usual to contact the N-terminus of D1-βC’, and CDR-H1 and -H3 are slightly moved away from each other to allow more space to accommodate the antigen β-strand, resulting in five hydrogen bonds between D1-βC’ and the heavy chain (Fig. 5c). Only one of these bonds occurs between a side chain group of D1-βC’ residues (Lys63-NH3^+^) and the 5A12 heavy chain. Moreover, the aliphatic chain of CD147 Lys63 participates in hydrophobic interactions with the aromatic side chains of Trp52 and Tyr120 from the 5A12 heavy chain, which, together with other hydrophobic interactions contributed by residues located at D1-βC’ (Val61, Leu62, and Glu64), further enhances the affinity between CD147 and 5A12. The binding of CD147-D1 to the light chain of 5A12 is obviously weaker than its binding to the heavy chain—only two hydrogen bonds exist between D1-βE and the light chain of 5A12, and no hydrophobic interactions were observed (Fig. 5d).

### Key residues in the CD147-5A12 mAb epitope and its function in Tm-cell activation

Having observed the major 5A12 epitope (61VLKEDA66) in the CD147_ecto_-5A12_Fab_ complex structure, we further aimed to identify which residue was the most important. First, five mutations were individually inserted in the wild-type CD147 by site-directed mutagenesis as follows: V61A, L62A, K63A, E64A, and D65A. The biophysical 5A12-binding parameters were determined using surface plasmon resonance (SPR) (Fig. 5e). Four of the individual reversion mutants (L62A, K63A, E64A, and D65A) showed decreased affinity, indicating that these mutations contribute favorably to 5A12. Among the four single mutations, K63A and D65A exhibited relatively lower binding affinity compared with the wild-type CD147. Then, we constructed the double mutation K63A/D65A, and its binding parameter (KD) decreased from 5.66 × 10^-10^ mol^-1^ to 1.15 × 10^-7^ mol^-1^, suggesting that Lys63 and Asp65 are the two most important residues in the 5A12 epitope.

To further verify whether mutations of K63A/D65A can exert similar inhibitory effects on Tm-cell activation as 5A12, the CD147 with the double mutation (MT) was transfected into Tm cells (Fig. 5f). FACS analyses showed that MT could inhibit Tm-cell activation, and the expression of the activation markers CD69 and CD25 was significantly reduced after both anti-CD3 (Fig. 5g) and anti-CD3/CD28 stimulation (Figs. 5h, i) compared with that of the revertant type (RT).

## Discussion

In this study, we demonstrated the aberrant upregulation of CD147 on the surface of CD4^+^ Tm cells from the peripheral blood and SF from RA patients and that the elevation of CD147 facilitates the hyper-activation of Tm cells and promotes osteoclastogenesis in RA. In addition, we found that mAb 5A12 is capable of inhibiting the ZAP70-mediated early activation of Tm-cell activation, impairing Tm-cell proliferation, and inhibiting osteoclastogenesis in vivo. Moreover, a structural biology study of the CD147_ecto_-5A12_Fab_ complex and structure-aided mutagenesis scanning revealed that the residues Lys63 and Asp65, two hotspots in the CD147-5A12 interacting interface, were critical for CD147**-**mediated co-stimulation in Tm cells.

Early in 1996, Neidhart et al. demonstrated that RA patients had elevated proportions of CD4^+^ T cells, which were correlated with the clinical parameters of disease activities.^24^ Subsequent studies gradually recognized an important role for Tm cells in the pathogenesis of autoimmune diseases such as RA, which was likely related to aberrant T-cell activation, leading to a surge in the number of Tm cells.^25–27^ In the present study, we found that RA patients manifested a predominantly high number of Tm cells in both SF and the peripheral blood. Meanwhile, increased expression of CD69 was observed on Tm cells from RA patients, indicating the continuous activation of Tm cells in RA, as circulating blood Tm cells were considered to uniformly lack CD69 expression in healthy individuals.^28^ Previous studies have identified a close association of activated T cells with the formation of osteoclasts, which were further involved in bone erosion during RA progression.^29^ In the present study, we found that it is the accumulated activated Tm cells in RA patients that play a more significant role, which further enhance osteoclastogenesis and promote subsequent bone destruction. Additionally, the blockade of CD147 by mAb on Tm cells significantly inhibited Tm-cell activation and further repressed osteoclastogenesis, indicating an important role for CD147 signaling in Tm-cell function and the pathogenesis of RA.

Numerous studies have shown that CD147 is widely expressed on a variety of cells, including lymphocytes. However, poor expression on resting T lymphocytes and high expression on activated T lymphocytes has been observed.^15, 28^ In this study, significantly higher expression of CD147 was observed in Tm cells than in Tn cells in either HD or RA patients. Upon activation, Tm cells also exhibited much greater upregulation of CD147 than Tn cells, suggesting a closer association between CD147 and Tm-cell activation. The evidence illustrates that CD147 plays a role in T-cell activation and proliferation. In a mouse model with a conventional gene-targeting strategy, Igakura et al. reported that CD147 knockout mice exhibited an enhanced mitogenic response of T lymphocytes in mixed lymphocyte reactions.^30^ Our previous in vivo study also showed stronger proliferation after stimulation with anti-CD3/CD28 antibodies in CD147 conditional knockout mice of CD4^+^ T cells.^31^ All these studies were performed in a mouse KO model with total T cells. The exact role of CD147 in human Tm cells is still not clear. In this study, human Tm cells were further studied. The activation of T cells was significantly inhibited either by silencing CD147 or blocking with a mAb. Additionally, a double-mutant CD147 (K63A/D65A) showed reduced affinity and inhibited T-cell activation, suggesting that CD147 has a co-stimulatory function by directly promoting TCR signaling.

Previous studies have consistently shown that CD147-specific antibodies to the extracellular region can block TCR-mediated lymphocyte activation and proliferation.^32, 33^ Animal experiments have also revealed that the inhibition of CD147 by anti-CD147 mAb alleviated inflammation in mouse models of acute lung inflammation, asthma, and RA.^20, 34–36^ These data supported a potentially promising application of CD147 mAbs in the treatment of inflammatory diseases. MEM-M6/6 is a mAb that recognizes the membrane proximal domain 2 of CD147, and it has been reported to decrease the proliferation of PBMCs and purified total CD3^+^ T cells.^33^ Another anti-CD147 antibody has also been shown to nonspecifically inhibit the activation of both CD45RA^+^ (naive) T cells and CD45RO^+^ (memory) T cells.^37^ In this study, we reported that 5A12, an anti-CD147 mAb established in our laboratory, could specifically inhibit TCR-stimulated Tm-cell activation and proliferation, whereas no inhibitory effect was found in Tn cells. Strategies designed specifically to suppress the function of chronically activated Tm cells without impairing the function of Tn cells therefore have value in the treatment of autoimmune diseases. The selective therapeutic mAb targeting of activated Tm cells directly involved in RA will definitely avoid the limitation of clinical application characterized by an increased susceptibility to infection and weakened cancer immunosurveillance with mAbs, which acted in a “pan” manner.^38^ On this basis, 5A12 was assumed to be an attractive agent to be used in RA.

The difference in antibody responses could be a result of differences in experimental methods (e.g., the time after T-cell activation) or perhaps a result of the different functional domains/epitopes of CD147 that the antibodies recognize and to which they bind. Crystallographic studies on the antigen–antibody complexes will definitely help understand the mechanisms of antigen function and benefit the design and development of novel targeting drugs.^39, 40^ By successfully resolving the crystal structure of the CD147_ecto_-5A12_Fab_ complex at a resolution of 2.6 Å, we identified ^61^VLKEDA^66^, β-strand βC’ in CD147-D1 as the major interaction epitope for 5A12, and the residues Lys63 and Asp65 were further identified as the most important via mutagenesis studies. Such structural information for the CD147-5A12 complex can probably provide a hint to predict the effect of the double mutation K63A/D65A. Additionally, the detailed effects of this epitope must be further investigated using different functional tests. Freshly isolated human Tm cells were transfected with CD147 short hairpin RNA (shRNA) lentiviruses and then re-expressed in a K63A/D65A mutant. In vitro experiments confirmed that this CD147 mutation could significantly inhibit Tm-cell activation as mAb 5A12 does, indicating that these two residues are critical for binding of 5A12 to CD147 in the regulation of Tm-cell activation.

**Fig. 6:**
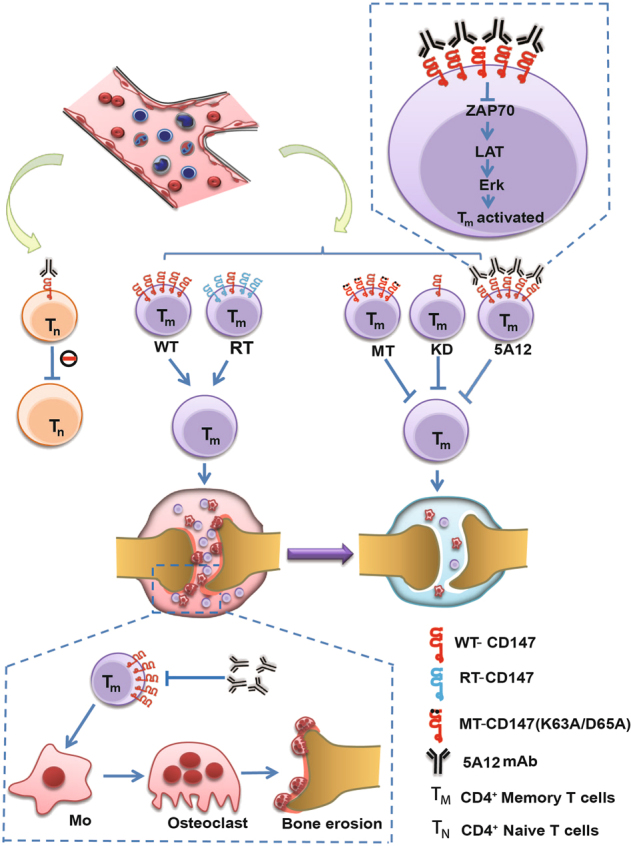
CD147-Tm/Osteoclast-RA chain. CD147 is predominantly upregulated on Tm cells from PBMCs of active RA patients. Anti-CD147 mAb 5A12 specifically inhibits Tm-cell activation and proliferation by decreasing ZAP70, LAT, and Erk phosphorylation and further restricts Tm-mediated osteoclastogenesis. Knockdown of CD147 (KD) or CD147 mutation (MT, K63A/D65A) on Tm cells exhibits similar inhibitory effects to 5A12, compared with a CD147 revertant (RT). Importantly, the K63D65 residues are the most important epitope in the CD147_ecto_-5A12_Fab_ complex and hence are a potential target for RA treatments

Our results allowed us to propose and provide evidence to support a “CD147-Tm/Osteoclast-RA chain” hypothesis, which is shown in Fig. 6. High expression of CD147 on Tm cells participated in abnormal Tm-cell activation and further promoted RA progression. This discovery will not only lay a solid foundation for a full understanding of RA pathogenesis but also provide a potential target of CD147 on Tm cells for RA treatment. Especially, mAb 5A12 exhibited specific inhibitory effects on Tm-cell activation and osteoclast formation. Its biological mechanisms of action are possibly related to the downregulation of the phosphorylation levels of ZAP70, LAT, and Erk. On the other hand, the mutations of two key residues, K63A/D65A, based on the crystallographically observed 5A12 epitope, also exert similar inhibitory effects on Tm-cell activation, which provides a promising target for designing structure-based small molecule drugs in T-cell**-**mediated autoimmune diseases.

## Materials and methods

### Ethics statement

Written informed consent was obtained from all patients and HDs, and the study was approved by the ethics committee of the Affiliated Xi Jing Hospital of the Fourth Military Medical University (XJYYLL-2012548).

### Patients

A total of 33 patients with active RA, fulfilling the criteria of the American College of Rheumatology, were enrolled in this study. The disease activity was assessed using the 28-joint disease activity score system (DAS28),^41^ and all patients were free of infectious diseases, malignant diseases, cardiovascular complaints, or other inflammatory diseases. The HDs consisted of 15 age- and sex-matched subjects with no symptoms of joint inflammation, no chronic inflammatory or autoimmune diseases, and no cancer in their personal or family history. HDs did not have current infections or allergic symptoms at the time of the study. The characteristics of RA patients and HDs are summarized in Table S1. Samples of the SF from the knee joints were obtained from four patients with active RA during surgery.

### T-cell subset separation

PBMCs were isolated from HDs and RA patients by Ficoll-density gradient centrifugation (Axis-Shield). To obtain enough peripheral blood cells for the mAb blocking, cell signaling and lentiviral knockdown experiments, 400 ml peripheral blood was collected from other HDs with no inflammation or cancers. The cells were washed three times with sterile phosphate-buffered saline (PBS) and suspended at a concentration of 2 × 10^6^ cells/ml in RPMI-1640 (GIBCO) supplemented with 10% heat-inactivated fetal calf serum, 2 mM l-glutamine, and 1% penicillin–streptomycin (HyClone). CD4^+^ T cells were isolated from PBMCs using a human CD4^+^T-cell negative-isolation kit (Miltenyi Biotec) according to the manufacturer’s instructions. CD4^+^CD45RO^+^ (Tm) and CD4^+^CD45RO^-^ (Tn) cells were isolated using a CD45RO^+^cell isolation kit according to the manufacturer’s manual (Miltenyi Biotec). The cells were routinely analyzed by flow cytometry, and the purity of both the Tm and Tn population exceeded 90%.

### Flow cytometry analysis

Antibodies were obtained from BD Pharmingen. The activation marker CD69 was analyzed to determine the activation levels of CD4^+^ T cells in RA. Samples of the peripheral blood and SF from RA patients and HDs were collected. The cells were incubated with phycoerythrin (PE)-conjugated anti-CD69 (FN50) and fluorescein isothiocyanate (FITC)-conjugated anti-CD25 (M-A251) on ice for 30 min. After washing with PBS three times, the cells were assayed using a four-color FACS Calibur flow cytometer (Becton Dickinson). To determine the CD147 expression levels in different peripheral blood cells, peripheral blood samples (100 μl) were stained for 30 min with the antibodies CD4‑FITC (RPA-T4), CD8‑FITC (RPA-T8), CD19‑FITC (HIB19), and CD14‑FITC (M5E2) (BD) to identify T cells, B cells, and monocytes, respectively,. Anti-CD147‑PE staining was used to determine CD147 expression. To test CD147 expression on Tcm and Tem cells, peripheral blood samples were stained with antibodies CD4‑APC-H7 (RPA-T4), CD45RO‑Percp-cy5.5 (UCHL1), CD45RA-PE-CY7 (5H9), and CD62L-BB515 (SK11) (BD) to identify Tcm, Tem, and Tn, respectively. Anti-CD147-AF647 (HIM6) was used to determine CD147 expression. The samples were assayed, and the Flowjo software was utilized for data analyses. The cell surface expression levels of CD69 and CD25 were also detected by the direct binding of a fluorescein-conjugated antibody as described above.

### Stimulation of Tm and Tn in vitro

Purified Tm and Tn were incubated in RPMI-1640 medium supplemented with 10% fetal bovine serum (FBS) and penicillin–streptomycin in the presence or absence of CD147 mAb 5A12 (10 μg/ml) for 1 h. The cells were then added into 96-well plates coated with anti-CD3 (0.5 μg/ml) (R&D) or/and anti-CD28 mAb (1 μg/ml) (R&D) at the indicated times. CD69 was examined 6 h after stimulation and CD25 24 h after stimulation by flow cytometry as previously described. For time-dependent experiments, the expression of CD69 and CD25 was determined at the indicated times after stimulation in vitro.

### Analysis of T-cell proliferation via CFSE staining

Both Tm and Tn were labeled with carboxyfluorescein diacetate succinimidyl ester (CFSE) (Invitrogen) according to the manufacturer’s instructions. The CFSE-labeled cells were then added to 96**-**well plates (1 × 10^5^ cells/well) and incubated in the presence or absence of 5A12 (10 μg/ml) at 37 °C for 1 h. Subsequently, anti-CD3/CD28 Dynal beads (Life Technologies) were added to each well at a ratio of 3 to 1 (beads to T cells). The cells were maintained in RPMI**-**1640 complete medium containing 10% FBS in a 37 °C incubator for 3 or 5 days, after which the T cells were harvested and analyzed using a FACS flow cytometer.

### T-cell signaling measurements

The total protein concentration of the cellular lysates was determined using a Luminex system. The corresponding levels of proteins involved in TCR signaling transduction, such as phosphorylated CREB (Ser133), Erk/MAP kinase 1/2 (Thr185/Tyr187), and phosphorylated tyrosine residues on the CD3 epsilon chain, LCK, ZAP70, LAT, and SYK, in T-cell supernatants were assayed using the MILLIPLEX®MAP7-Plex Human T-Cell Receptor (TCR) Magnetic Bead Kit (Merck Millipore) according to the manufacturer's instructions and acquired on a Bio-Plex 200 flow-based sorting and detection analyzer (Bio-Rad). The results for all of the experiments were expressed as the average of triplicate tests.

### Cytokine multiplex assay

Serum samples were collected from both RA patients and HDs for a cytokine multiplex analysis. Tm cells were also preincubated with 5A12 and isotype for 1 h and then stimulated for 24 h with anti-CD3/CD28 mAbs. Cytokines in the different groups were detected by Luminex. Cytokine production was measured with the Luminex panel (IFN-γ, TNF-α, IL-1β, IL-2, IL-4, IL-6, IL-12, IL-17A, IL-21, IL-22, and IL-23) (Merck Millipore) using a Bio-Plex 200 flow-based sorting and detection analyzer (Bio-Rad).

### Lentiviral CD147 knockdown

To knockdown the expression of CD147 in primary Tm cells, we used the Trans-Lentiviral pLKO System to package the lentivirus. The experiments were performed as previously described.^42^ Isolated Tm were then cultured in 24-well plates precoated with anti-CD3 (1 μg/ml) and anti-CD28 (5 μg/ml) in the presence of human recombinant IL-2 (R&D) (20 U/ml) for 24-h preinfection. The viral infection was carried out by centrifugation at 2000 rpm at 4 °C for 120 min. Then, the cells were incubated for 48 h at 37 °C in the presence of the viral supernatant. Fresh medium was added after the virus was removed. The infected cells were selected for puromycin (Sigma-Aldrich) resistance in a medium containing 2 μg/ml puromycin for 2 days; after the selection, the cells were harvested for subsequent experiments after IL-2 withdrawal for 48 h.

### In vitro induction of osteoclasts

Human PBMCs were collected from HDs and separated by gradient centrifugation. The CD14^+^monocytes were then isolated as osteoclast precursors using a human CD14-positive isolation kit (Miltenyi Biotec) and cultured in alpha-minimal essential medium supplemented with 10% FBS stimulated with 20 ng/ml recombinant M-CSF (R&D) for 6 days. After 6 days, the preosteoclast cells were further cultured in the presence of 40 ng/ml M-CSF and 20 ng/ml human RANKL (R&D) for 5 days, and fresh solutions were added every 3 days. Mature osteoclasts were stained for tartrate-resistant acid phosphatase (TRAP) using a leukocyte acid phosphatase kit (Sigma) according to the manufacturer’s instructions, and the TRAP-positive multinucleated cells were counted in each well.

To investigate the possible role of Tm cells in the differentiation of osteoclasts, we isolated monocytes and cultured them with Tm and Tn cells under activation conditions to observe the osteoclastogenesis. Tm and Tn cells isolated from HDs were plated in 96-well plates coated with anti-CD3 mAb (5 μg/ml) and medium containing anti-CD28 mAb (0.2 μg/ml) at 2 × 10^4^ cells per well for 2 days before co-culture with osteoclast precursors. The positive control was CD14^+^ monocytes incubated with M-CSF (40 ng/ml) and RANKL (20 ng/ml). All experiments included unstimulated (“resting”) T-cell controls. To further study whether CD147 is involved in Tm-mediated osteoclastogenesis, both Tm and Tn cells were isolated and pretreated with CD147 mAb 5A12 before activation, and then, the cells were co-cultured with CD14^+^ monocytes.

### Preparation of the CD147_ecto_-5A12_Fab_ complex

CD147_ecto_ was recombinantly expressed in *Escherichia coli*and purified as we described previously.^43^ The 5A12 mAb was prepared as previously reported^44^ and then cleaved by papain (5A12:papain (w/w) = 600:1) at 37 °C for 8 h. The papain-cleaved 5A12 was applied on a HiTrap MabSelect SuRe column to remove the Fc fragments. The 5A12_Fab_ in the eluent was further purified by size exclusion chromatography in a solution of 20 mM sodium phosphate, 0.15 M NaCl, pH 7.2. The purified CD147_ecto_ and 5A12_Fab_ were incubated overnight at 4 °C at a molar ratio of 1:1.5 (5A12_Fab_:CD147_ecto_). The mixture was applied to a Superdex 200 10/300 (GE Healthcare) column to perform size exclusion chromatography. The purified CD147_ecto_-5A12_Fab_ complex was then concentrated for further analysis.

### Crystallization, data collection, and structure determination

The purified CD147_ecto_-5A12_Fab_ complex was co-crystallized at 10 °C via the hanging drop vapor diffusion method, and complex crystals were successfully obtained in the presence of 0.2 M magnesium acetate and 10% (w/v) PEG8000. X-ray diffraction data of the complex crystals were collected at 100 K at the beamline BL17U1 of the Shanghai Synchrotron Radiation Facility (Shanghai, China) at a wavelength of 0.97923 Å. A 2.6-Å resolution dataset was integrated and scaled using the HKL2000^32^ software. The phasing problem was solved by using molecular replacement program PHENIX.Phaser^45^ with the previously determined crystal structures of CD147_ecto_ (PDB entry: 3B5H)^43^ and the mouse antibody Fab (PDB entry: 5H90) as the search models. The initial model containing six CD147_ecto_ and six 5A12_Fab_ was refined by iterative rounds of manual adjustment in COOT^46^ and subsequently refined with PHENIX.Refine.^47^ The statistics for the data collection and structure refinement are listed in Table S2.

### Construction of CD147 mutants

We previously developed a CD147/pEGFP-N1 vector that encodes the CD147 protein^48^ and used it as a template for CD147 mutant construction in the present study. The QuickChange Site-Directed Mutagenesis kit (Stratagene) was used to generate a site-directed mutant of the 5A12-binding epitope of CD147 from 61 to 65 according to the manufacturer’s instructions. The sequences of the PCR products were confirmed by sequencing by Shanghai Sangon Co. (Shanghai, China). The identified mutant vectors were then inserted into pET21a (+) (Novagen) with *Nde*I and *Xho*I, and their integrity was confirmed by automated sequencing. These constructs were chemically transformed into Origami B and grown in luria-bertani medium, yielding the secretion of CD147 mutants. Purification was done using the AKTA fast protein liquid chromatography system.

### SPR measurements

The binding kinetics and affinity of 5A12 for the purified CD147 protein were obtained using the ProteON XPR36protein interaction array system (Bio-Rad). 5A12 was immobilized onto a GLC chip. One flow channel was activated in parallel with 10 mM EDAC and 40 mM sulfo-NHSat 25 μl/min for 5 min. 5A12 was diluted to 10 μg/ml in 10 mM sodium acetate pH 5.5 and directly coupled onto the activated channel for 5 min. Excess reactive esters were blocked with a 5-min injection of 1 M ethanolamine HCl. The mean immobilization level was 2100 RU. One channel was left unmodified to provide an additional reference surface. Wild-type and mutated CD147 proteins were prepared as twofold serial dilutions (0, 0.625, 1.25, 2.5, 5, and 10 nM) and injected for 180 s at 50 μl/min. Dissociation was monitored for 720 s. The surface was regenerated with multiple 15-s pulses of 10 mM Gly-HCl so that the experiments could be reproduced. Then, the binding kinetics of CD147 and mutations of 5A12 were determined using a “one-shot” kinetic mode.

### Re-expression of mutants

To further test the functional effects of the CD147 mutants on Tm-cell activation, the rescue of the pre-knockdown phenotype by RNA interference (RNAi)-resistant gene re-expression was a critical validation step. CD147 mutant lentiviral vectors that encoded an altered mRNA, which is resistant to siRNA silencing (one with synonymous mutations at the shRNA target site), were designed by GeneChem (Shanghai, China). Tm cells were then transfected with the mutant lentivirus and revertant lentivirus on the basis of CD147 downregulation, and the detailed procedures were similar to “Lentiviral CD147 knockdown”.

### Statistical analysis

All of the data were expressed as the mean ± SD and were obtained from at least three replicate observations. Statistical procedures were performed using the GraphPad software Prism-6 (La Jolla, CA). A one-way analysis of variance and a *t*-test were used to compare the differences between groups.

## Electronic supplementary material

supplementary data
