# Supplementary material for: A critical epitope in CD147 facilitates memory CD4+ T-cell hyper-activation in rheumatoid arthritis
Source: Cell Mol Immunol. 2018 Mar 21;16(6):568–79. doi: 10.1038/s41423-018-0012-4 (PMC6804595; doi:10.1038/s41423-018-0012-4)
Supplement: Supplementary file 1 — supplementary data [file 41423_2018_12_MOESM1_ESM.docx]

**Supplemental Information**

**Critical epitope in CD147 facilitating memory CD4+ T cell hyper-activation in rheumatoid arthritis**

**Na Guo, Kui Zhang, Sheng Ye, Xiaoling Yu, Hongyong Cui, Xiangmin Yang, Peng lin, Minghua Lv, Jinlin Miao,Qing Han, Rongguang Zhang, Zhinan Chen, Ping Zhu**

**Supplementary Figure 1**

**
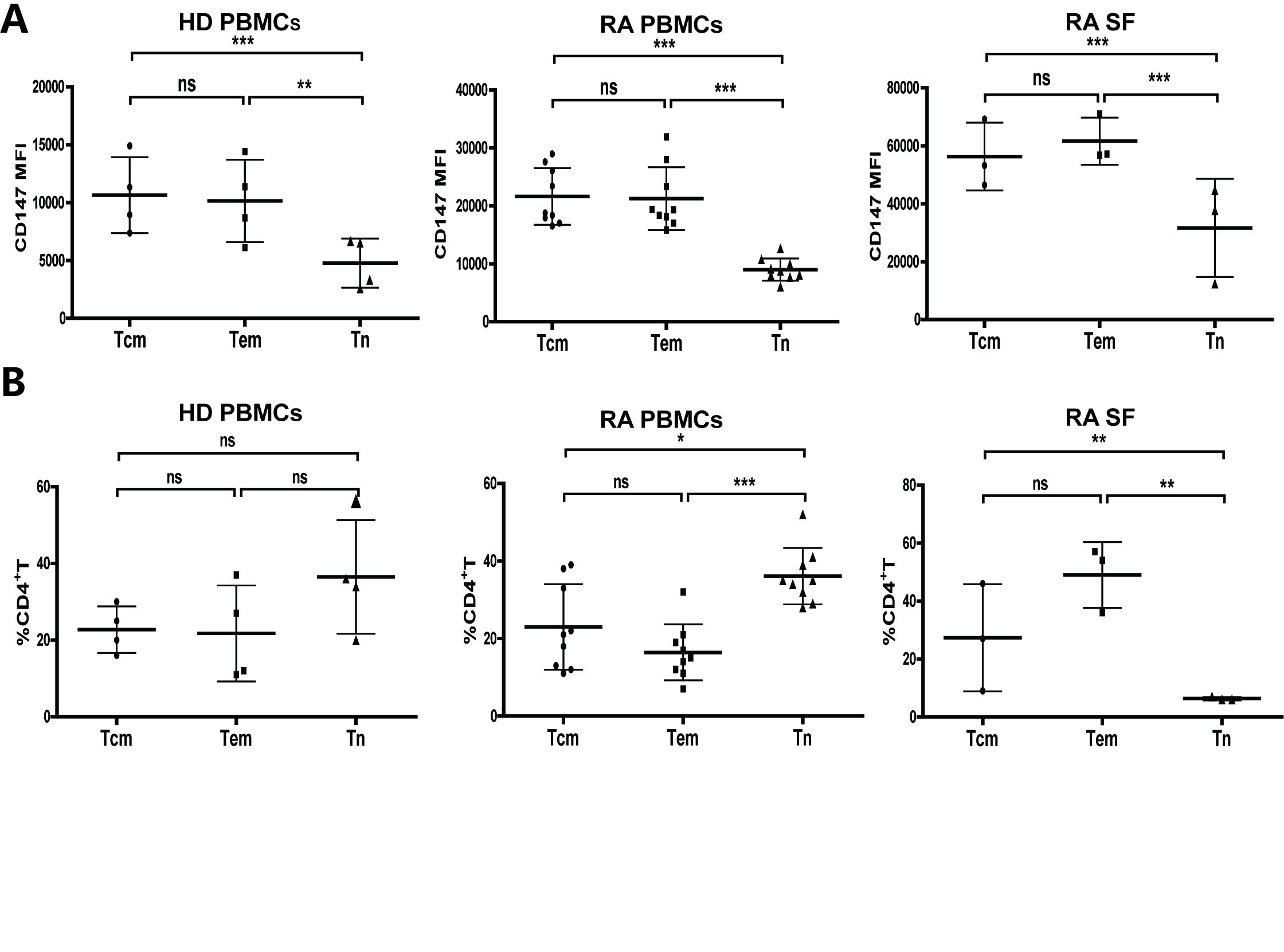
**

**Supplementary Figure 1. *CD147 expression showed no significant difference between Tcm and Tem.*** (A) CD147 expression on Tcm, Tem and Tn in PBMC of both HD (n=5) and RA patients (n=10) was tested by flow cytometry. 3 cases of SF from RA patients were also collected to test CD147 expression on Tcm, Tem and Tn cells. (B) The proportions of Tcm, Tem and Tn in CD4+ T cells were also tested in HD (n=5) and RA patients (n=10). *, P<0.05; **, P<0.01; ***, P<0.001; ns, no significance.

**Supplementary Figure 2**

**
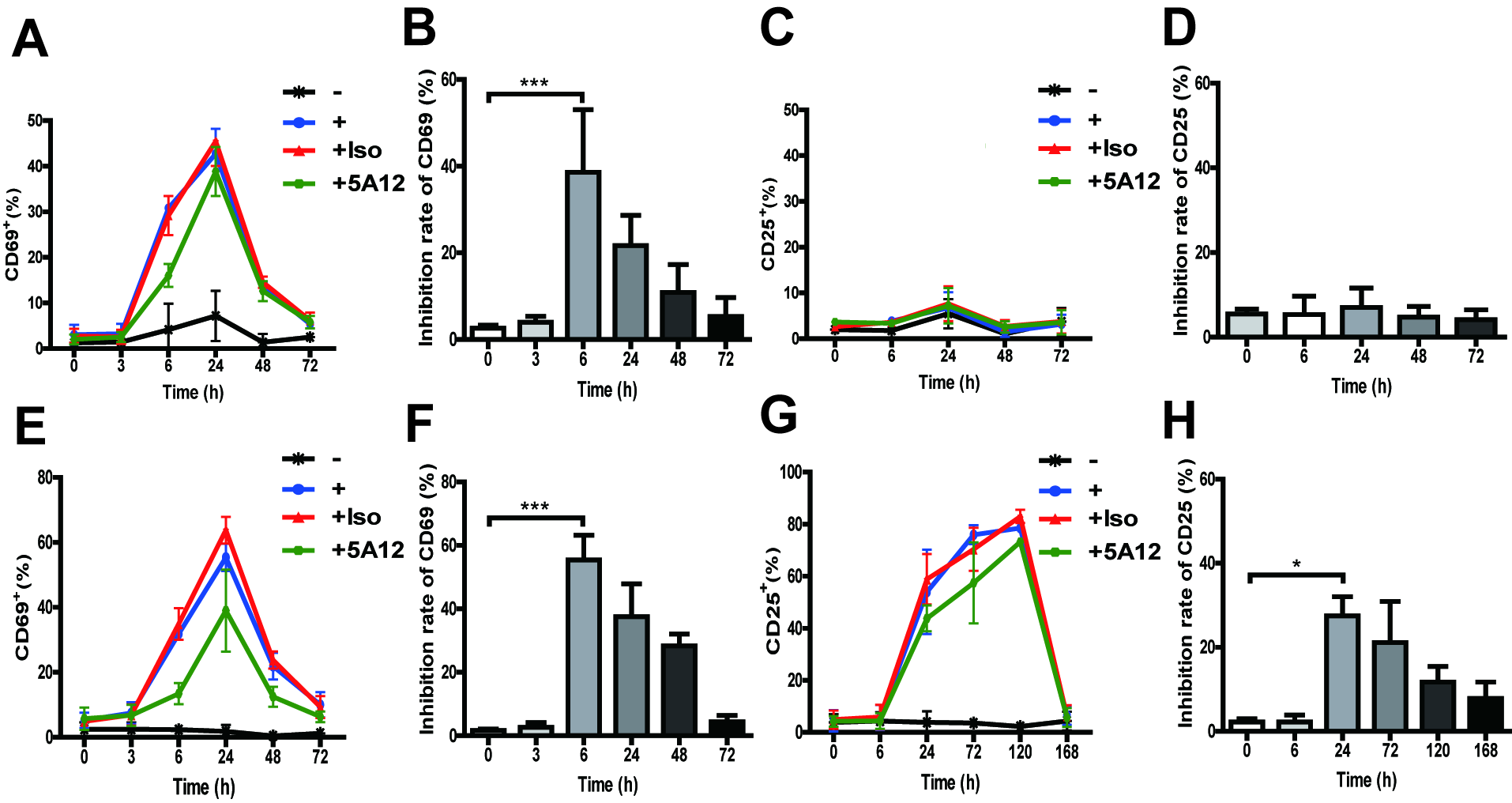
**

**Supplementary Figure 2. *Inhibition of Tm activation by 5A12 in a time-dependent manner.*** (A and C) The Percentages of CD69+ and CD25+ memory T cells were analyzed by FCAS at indicated times after a single stimulation of anti-CD3mAb with or without 5A12 treatments. (B and D) The corresponding statistical results of inhibition rate were shown. (E and G) The Percentages of CD69+ and CD25+ memory T cells were analyzed by FCAS at indicated times after a double stimulation of anti-CD3/CD28mAbs with or without 5A12 treatments. (F and H) The corresponding statistical results of inhibition rate were shown. *, P<0.05; ***, P<0.001.

**Supplementary Figure 3**


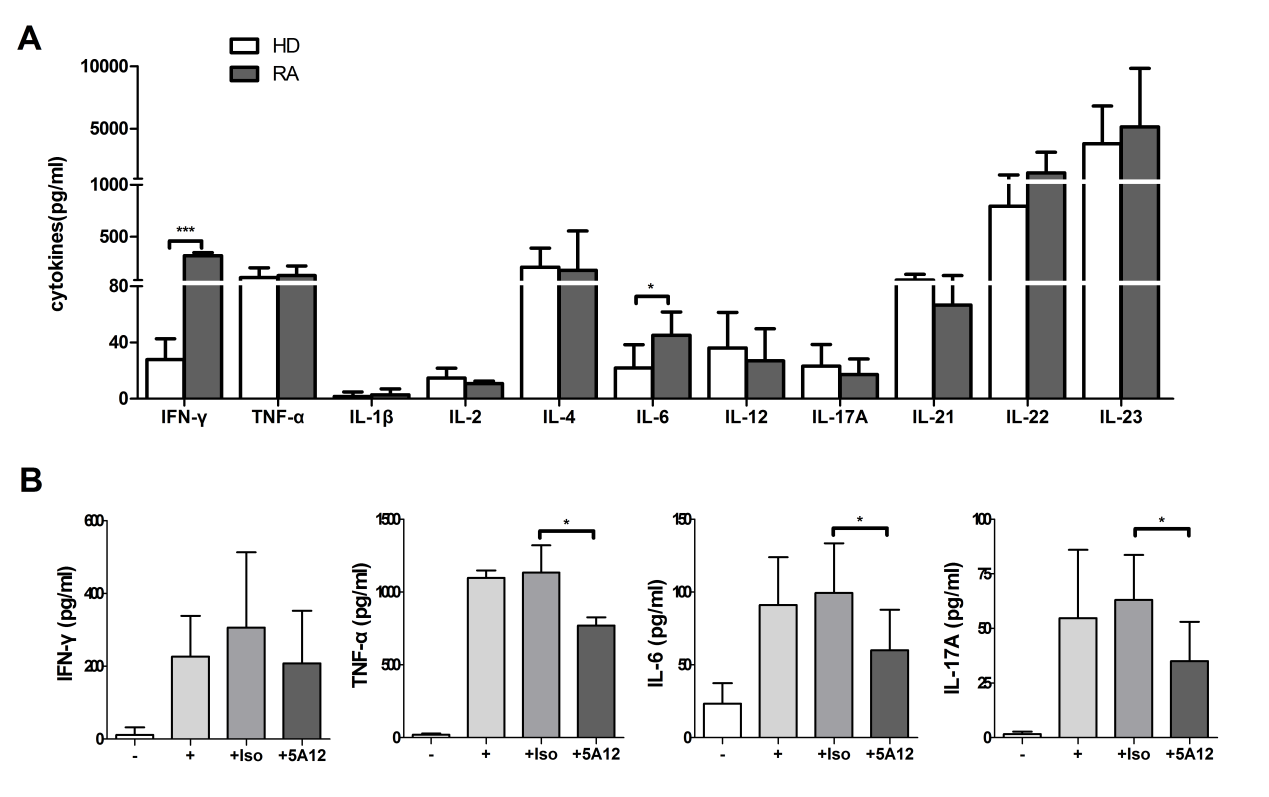


**Supplementary Figure 3. *5A12 significantly inhibited cytokine secretion upon Tm cell activation.***

(A) Cytokine production of periphearal blood serum from RA (n=10) and HD (n=5) was measured by Luminex. (B) Untreated Tm and Tm preincubated with 5A12 and Isotype for one hr and then stimulated for 24 hrs with anti-CD3/CD28 mAbs. Cytokines in different groups were detected by Luminex. *, P<0.05; ***, P<0.001.

**Supplementary Table 1**

**Table 1 Characteristics of RA patients and healthy donors**

| **Demographics** | **Active RA**  **(n=33)** | **Healthy donors**  **(n=15)** |
| --- | --- | --- |
| Age(years), median (IQR) | 37.0 (28.0-43.0) | 32.0 (28.0-43.5) |
| Sex, male/female | 7/25 | 5/10 |
| ESR (mm/h), median (IQR) | 30.0 (21.0-58.0) | NA |
| CRP(mg/L), median (IQR) | 2.72 (0.32-5.18) | NA |
| DAS28, median (IQR) | 4.72(3.91-5.40) | NA |
| Disease duration (months),  median (IQR) | 9.0 (3.0-12.0) | NA |

ESR: Erythrocyte sedimentation rate, CRP: C-reactive protein, DAS28: Disease Activity Score in 28 joints.

**Supplementary Table 2**

**Table 2 Crystallographic data collection and refinement statistics**

|  | **5A12:CD147** |
| --- | --- |
| **Data colleciton** |  |
| Resolution(Å) | 2.6(2.67-2.60) |
| X-ray wave length(Å) | 0.97923 |
| Total No. reflections | 143913 |
| Redundancy | 5.6(5.5) |
| *I*/σ*I* | 7.9(2.7) |
| Completeness(%) | 99.3(97.6) |
| *R _merge_* ^a^ | 0.114(0.610) |
| **Refinement** |  |
| Resolution(Å) | 48.7-2.6 |
| Space group | P2_1_ |
| Cell dimensions |  |
| *a, b, c* (Å) | 110.4, 127.5, 171.0 |
| *β(*˚) | 95.6 |
| No. reflections in working set | 136684 |
| No. reflections in test set | 7229 |
| *R_cryst_*^b^ | 0.209 |
| *R_free_*^c^ | 0.262 |
| Bond lengths( Å) | 0.003 |
| Average temperature factor | 59.8 |
| No. atoms(Water) | 1020 |

^a^R_merge_=(Σ│Ii-<Ii>│)/ Σ│Ii│, in which Ii is a given integral intensity of the diffraction spot; ^b^R_cryst_=(Σ││F_o_│-│F_c_││)/ Σ│F_o_│, in which F_o_andF_c_ denote the measured and calculated structure factor, respectively;

^c^R_free_ was calculated in the same manner with R_cryst_, but based on a 5% correction data from a randomly selected sample set.
